# Molecular Exploration of the First-Century *Tomb of the Shroud* in Akeldama, Jerusalem

**DOI:** 10.1371/journal.pone.0008319

**Published:** 2009-12-16

**Authors:** Carney D. Matheson, Kim K. Vernon, Arlene Lahti, Renee Fratpietro, Mark Spigelman, Shimon Gibson, Charles L. Greenblatt, Helen D. Donoghue

**Affiliations:** 1 Paleo-DNA Laboratory, Lakehead University, Thunder Bay, Canada; 2 Department of Anthropology, Lakehead University, Thunder Bay, Canada; 3 Department of Microbiology and Molecular Genetics, The Hebrew University-Hadassah Medical School, Jerusalem, Israel; 4 Department of Anthropology, Department of Zoology, University of Queensland, St. Lucia, Australia; 5 Department of Biology, Lakehead University, Thunder Bay, Canada; 6 Department of Infection, University College London, London, United Kingdom; 7 University of North Carolina at Charlotte, Charlotte, North Carolina, United States of America; University of Utah, United States of America

## Abstract

The Tomb of the Shroud is a first-century C.E. tomb discovered in Akeldama, Jerusalem, Israel that had been illegally entered and looted. The investigation of this tomb by an interdisciplinary team of researchers began in 2000. More than twenty stone ossuaries for collecting human bones were found, along with textiles from a burial shroud, hair and skeletal remains. The research presented here focuses on genetic analysis of the bioarchaeological remains from the tomb using mitochondrial DNA to examine familial relationships of the individuals within the tomb and molecular screening for the presence of disease. There are three mitochondrial haplotypes shared between a number of the remains analyzed suggesting a possible family tomb. There were two pathogens genetically detected within the collection of osteological samples, these were *Mycobacterium tuberculosis* and *Mycobacterium leprae*. The Tomb of the Shroud is one of very few examples of a preserved shrouded human burial and the only example of a plaster sealed loculus with remains genetically confirmed to have belonged to a shrouded male individual that suffered from tuberculosis and leprosy dating to the first-century C.E. This is the earliest case of leprosy with a confirmed date in which *M. leprae* DNA was detected.

## Introduction

**Figure 1:**
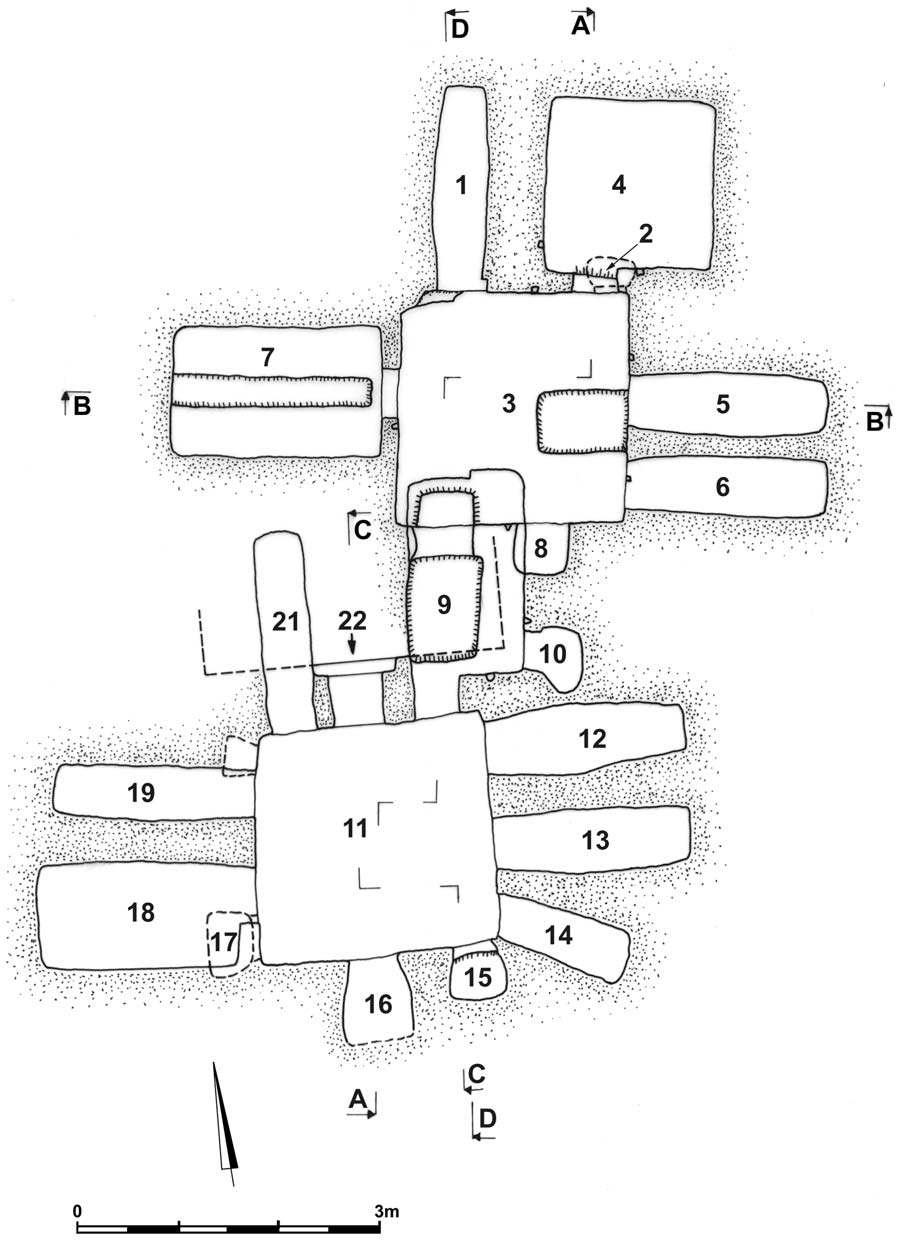
A schematic plan of the tomb (numbers represents the niche or loculi) (Produced by SG).

The Tomb of the Shroud was investigated in 2000 in the lower Hinnom Valley at the foot of Mount Zion by an archaeological team led by Shimon Gibson, Boaz Zissu and James Tabor [1], [2]. The tomb had been entered and looted before archaeologists reached the site. The tomb was one of more than seventy separate family tombs of the first century C.E. in an area identified with traditional Akeldama, the “Field of Blood” described in the Bible (Matthew 27:3–8; Acts 1∶19). The tomb comprised a simple entrance cut into a quarried scarp leading to rectangular rock-hewn chambers on two levels, with tunnel-like *kokhim* in the walls and with a number of niche repositories - *ossilegia* is the custom of bone collection in rock-hewn tombs - for the secondary storage of human remains (Figure 1). More than twenty stone ossuaries for the collection of human bone were found, a few bearing inscriptions in Jewish and Greek script (“Mary”; “Shimon ben [son of] Shulai”; “Salome”; “Phineas”). What marked this tomb as unique from the other tombs in the Akeldama cemetery was the discovery of degraded shroud textile, with a simple weave and a clump of human hair, in one of the loculi together with the skeletal remains of a primary burial of an adult. This discovery is rare because high levels of humidity in the Jerusalem area does not usually allow for the preservation of organic material. A radiocarbon date of the shroud textile determined by the AMS radiocarbon laboratory in Texas (by D. Donahue) confirmed its early date: 2025+/− 28 years BP, with calibrated calendar date ranges of one sigma: 50 BCE -16 CE and two sigma: 95 BCE -53 CE.

**Figure 2:**
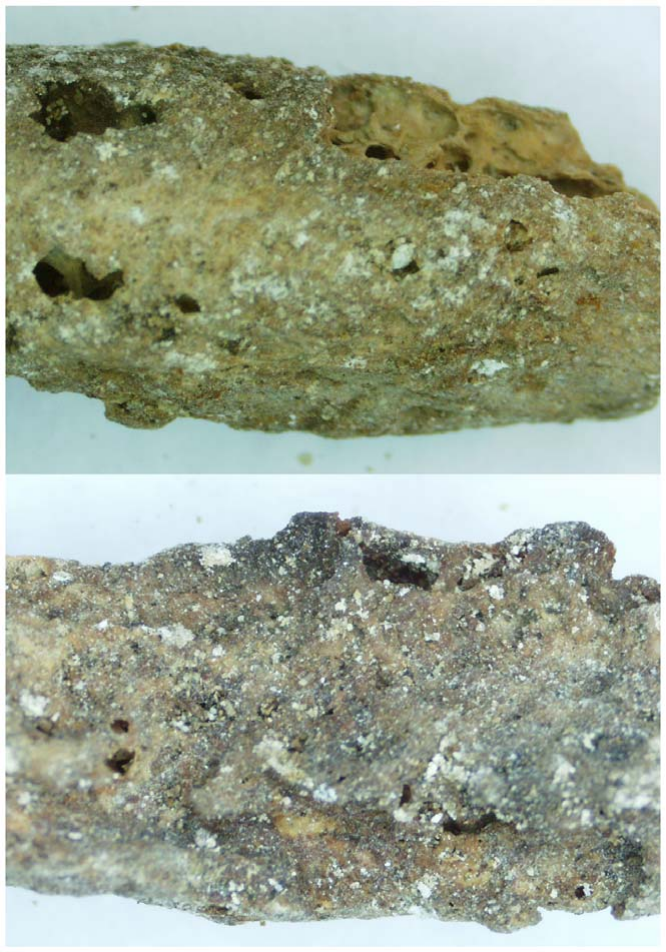
The phalanx with suspected pathology (Photograph taken by KKV).

The investigation of this tomb has included many areas of archaeological investigation and the genetic analysis of the bioarchaeological material from the Tomb of the Shroud was an integral aspect of this interdisciplinary analysis. The genetic analysis was performed because no other first century tomb from Jerusalem has ever been examined by molecular methods and this particular tomb has remains that are unique within this region - specifically the discovery of textiles that form part of a burial shroud from the first century. Also, due to the extremely disturbed nature of the tomb, extensively damaged osteological remains and recovery of less than 5% of each skeleton, traditional morphological analysis did not yield any information (MNI, height, age, sex, ancestry and pathology). The morphological analysis did identify one osteological element, a phalanx (from the hand), that presented with secondary osseous remodeling lesions, however these features were not pathognomonic for any disease (Figure 2). Since molecular analysis has proven to be successful for the detection of *Mycobacterium tuberculosis* and *Mycobacterium leprae* and these pathologies could be included in the differential diagnosis of this osteological element, all the samples in the tomb were screened for the presence of DNA from these two pathogens. Mitochondrial DNA (mtDNA) was analyzed to examine familial relationships of the individuals within the tomb, in parallel with the molecular paleopathology that identified the presence of disease. Genetic analysis involves a method termed polymerase chain reaction (PCR) for amplification and analysis of trace amounts of DNA, including pathogenic bacterial DNA, recovered from past human material and has been used successfully to identify and characterize ancient DNA. The recovery of ancient or degraded DNA is challenging and hindered by forms of damage, including chemical modifications and fragmentation of the DNA [3], [4], [5]. The mtDNA molecule has been used extensively for population analysis and the identification of maternal relationships. MtDNA is favoured over genomic DNA, with good recovery due to its high copy number that is over a thousand copies within each cell [3], [6], [7], [8], [9], [10]. Mitochondria are maternally inherited organelles, suitable for characterization of maternal relationships [11], [12], the traditional approach in familial genetics, identifying haplotypes and population groups [10], [13], [14], [15].

The first pathogenic DNA recovered from archaeological remains was *M. tuberculosis* [16]. This was quickly followed by the first report of *M. leprae* DNA in human remains from the seventh century C.E. [17], [18]. With recent advances in molecular detection of pathogens, research has focused on historically documented diseases and epidemics, although reports are still dominated by analyses of *M. tuberculosis* [19], [20], [21], and of *M. leprae*, the causative agent of leprosy (Hansen's disease) [22], [23], [24]. The preservation and persistence of ancient bacterial DNA in the archaeological record is controversial [25], however research into the mechanisms bacteria use for self preservation during periods of sub-optimal environmental conditions has provided supporting evidence for the long term preservation [26] and stability of DNA in bacterial pathogens.

Historically disfiguring diseases, particularly leprosy and tuberculosis, were commonly categorized together in the Near East and the afflicted individuals were ostracized from their communities. The general Jewish practice in the first century C.E. was for a primary burial to be placed within a loculus until the decomposition of organic remains had taken place, at which point - approximately a year later - the bones were then taken out of the loculus and transferred into a repository (a pit or wall niche) or into a stone ossuary. However this transfer did not occur for the individual buried in Tomb of the Shroud loculus 1 - instead this loculus was sealed with white plaster, a practice which is quite rare in the first century tombs studied around Jerusalem.

## Materials and Methods

Permission for this research was provided by the Israeli Antiquities Authority and the archaeologists who excavated the material. Standard molecular archaeology protocols were applied for analysis of the Tomb of the Shroud samples at the ancient DNA laboratory in the Kuvin Centre for Tropical and Infectious Diseases, Hebrew University (Israel) and replicated at the Paleo-DNA Laboratory, Lakehead University (Canada) and at the Department of Infection, University College London (UK).

### Archaeological Sample Selection

In this study of the Tomb of the Shroud (SC) there were 23 samples tested for genetic relatedness or similarity based on the mtDNA and disease, in addition three environmental matrix controls of sediment that had built up in the rock hewn tomb (Figure 1).

Microscopic examination of organic remains from the tomb identified at least one piece of bone supporting uncharacteristic osseous lesions that are not a product of the taphonomic environment. The identification of the causative agent for the lesion is hindered by the disturbed nature of the skeletal remains (caused by the illegal looters of the tomb), and limited preservation of skeletal elements, especially those in association with the affected region. Tuberculosis is the primary candidate for causing these lesions, and was the molecular focus of the pathological analysis. Pathological lesions and bone resorption of the phalanges typical for leprosy, was not observed in the few phalanges recovered from the shroud loculus (SC1), however, the co-infection of two mycobacterial diseases is not uncommon and has been previously identified [27]. Skeletal remains from numerous loculi were analyzed for the presence of *M. tuberculosis*. Teeth, textile and bone samples were selected, with seven samples from the shroud loculus (selected from the length of the loculi, SC1) to maximize the chance of analyzing bone infected with a pathogenic disease. Sediment samples from around the tomb and scrapings from the rock walls and loculus roof were analyzed for the presence of any pathogenic mycobacterial DNA or environmental DNA causing non-specific results.

### Genetic Analyses

#### Laboratory 1: The ancient DNA laboratory at the Kuvin Centre for Tropical and Infectious Diseases, Hebrew University, Israel

The extraction of DNA from the bone samples used prepared aliquots of the sample. These were first placed under UV irradiation with 254 nm wavelength for 24–48 hours, depending on the size of the sample, to crosslink any surface contaminating DNA from soil bacteria or handling. To ensure there was no contamination, bone material from inside the bone was removed by a dental pick while the surface of the bone was left intact. The removed bone particles were placed into a mortar and pestle, ground into a fine powder then placed into a sterile tube for extraction. The working area was bleached and washed with 3.0% (w/v) sodium hypochlorite solution followed by 70% ethanol before and after each sample preparation and all equipment was sterilized before use. The equipment and the work area were UV-irradiated prior to each preparation. The researchers adhered to all standard ancient DNA and forensic protocols including the use of laboratory coats, sleeves, double gloving and face mask with frequent changes of gloves.

The prepared sample, approximately 25 mg was placed in a 1.5 ml tube containing 500 µl of guanidine thiocyanate (GuSCN) solution (4.0 M GuSCN, 0.1 M tris-HCl pH 6.4, 20 mM EDTA pH 8.0, 1.3% Triton X-100) and vortexed for 1 minute then incubated at 56°C overnight under gentle agitation (modified from Boom *et al*. 1990 [28]). The following day the preparation was heated at 94°C for 10 minutes, centrifuged (3 minutes at 15,000 x g) and the supernatant transferred to another sterile 1.5 ml tube. Guanidine thiocyanate solution (1.0 ml) and silica bead (Sigma) suspension (10 µl), prepared as outlined by Boom *et al*. 1990 [28], were added to the sample. The tube was mixed for 20 seconds and placed on ice for 1 hour with agitation every 15 minutes. The sample was centrifuged at 15,000 x g or 30 seconds and the supernatant discarded carefully. Next, 500 µl of washing buffer (2.0 mM tris-HCl pH 7.5, 10 mM EDTA pH 8.0, 10 mM NaCl, 50% (v/v) water, 50% (v/v) ethanol) was added, mixed and centrifuged (30 seconds at 12,000 x g). The supernatant was discarded. The wash buffer step was repeated, if the pellet was discolored. The silica pellet was washed with 200 µl absolute ethanol as above and allowed to air dry. DNA was eluted with 150 µl of sterilized water, mixed for 20 seconds and incubated at 56°C for at least 1 hour then resuspended in sterilized water. The sample was centrifuged (3 minutes at 15,000 x g) and the DNA extract stored at 4°C. The whole procedure was performed with extraction negatives to ensure that no contamination had entered the samples during this process. Standard precautions to minimize contamination were employed, sterile tubes and plugged tips under UV hoods where PCR reagent preparations were physically separated, from both sample preparation and PCR, in separate rooms with separate air supply. All equipment and surfaces were washed with a 3.0% (w/v) sodium hypochlorite solution and UV irradiated at 254 nm wavelength between each use.

The PCR was in a final volume of 25 µl (consisting of: 200 mM tris-HCl pH 8.4, 500 mM KCl, 2.0 mM MgCl_2_, 2.5 mM of each nucleotide (dATP, dCTP, dGTP and dTTP), 0.25 µM of each primer, 1.25 units of Platinum *Taq* DNA polymerase (Invitrogen). The DNA extract (10.0 µl) was added to each tube and both negative and positive controls were always included. The positive control DNA was diluted to simulate ancient DNA concentrations and was added to the positive control reaction tubes in a separate room from where the PCR reactions were prepared, before being loaded onto the thermal cycler. The amplification program consisted of a 3 minutes initial denaturation at 94°C, followed by 45 cycles of: 94°C for 1 minute, 60°C for 1 minute and 72°C for 2 minute; followed by 10 minute at 72°C. Mitochondrial DNA primers for the Hypervariable region 1 (HV1) were used [29], [30] and sexing primers [31]. Primers were designed for the analysis of Hypervariable region 2 (HV2) (Table 1). Molecular analysis for sex identification was performed because the remains were too incomplete and fragmented to identify the sex of the individuals through morphological analysis.

**Table 1:** The mitochondrial DNA, sex and disease primers used in this study.

Organisim	Name	Target region	Primer Sequence (5′ – 3′)	Reference
***H. sapiens***	mt15975F	mtDNA Hypervariable Region 1	CTCCACCATTAGCACCCAAAGC	[29]
	mt16190F	mtDNA Hypervariable Region 1	CCCATGCCTACAAGCAAGTA	[51]
	mt16322R	mtDNA Hypervariable Region 1	TGGCTTTATGTACTATGTACTG	[30]
	mt16420R	mtDNA Hypervariable Region 1	TGATTTCACGGAGGATGGTG	[29]
	mt1F	mtDNA Hypervariable Region 2	GATCACAGGTCTATCACCC	[36]
	mt15F	mtDNA Hypervariable Region 2	CACCCTATTAACCACTCACG	[52]
	mt155F	mtDNA Hypervariable Region 2	TATTTATCGCACCTACGTTC	[36]
	mt279R	mtDNA Hypervariable Region 2	GATGTCTGTGTGGAAAGTGG	[36]
	mt429R	mtDNA Hypervariable Region 2	CTGTTAAAAGTGCATACCGCCA	[29]
	Amel1	Amelogenin gene	CCCTGGGCTCTGTAAAGAATAGTG	[31]
	Amel2	Amelogenin gene	ATCAGAGCTTAAACTGGGAAGCTG	[31]
	ARXF	X chromosome Alphoid Repeat	AATCATCAAATGGAGATTTG	[53]
	ARXR	X chromosome Alphoid Repeat	GTTCAGCTCTGTGAGTGAAA	[53]
	ARYF	Y chromosome Alphoid Repeat	ATGATAGAAACGGAAATATG	[53]
	ARYR	Y chromosome Alphoid Repeat	AGTAGAATGCAAAGGGCTCC	[53]
***M. tuberculosis***	INS.1	IS*6110* repeat element	CGTGAGGGCATCGAGGTGGC	[32]
	INS.2	IS*6110* repeat element	GCGTAGGCGTCGGTGACAAA	[32]
	P1	IS*6110* repeat element	CTCGTCCAGCGCCGCTTCGG	[33]
	P2	IS*6110* repeat element	CCTGCGAGCGTAGGCGTCGG	[33]
	IS3	IS*6110* repeat element	TTCGGACCACCAGCACCTAA	[20]
	IS4	IS*6110* repeat element	TCGGTGACAAAGGCCACGTA	[20]
	S12.F	Ribosomal protein S12 gene	CGTCGGGACAAGATCAGTAAG	[54]
	S12.R	Ribosomal protein S12 gene	CAACCTGCAGGAGCACTCGAT	[54]
***M. leprae***	LP1	RLEP repetitive element	TGCATGTCATGGCCTTGAGG	[24]
	LP2	RLEP repetitive element	CACCGATACCAGCGGCAGAA	[24]
	LP3	RLEP repetitive element	TGAGGTGTCGGCGTGGTC	[24]
	LP4	RLEP repetitive element	CAGAAATGGTGCAAGGGA	[24]
	LP5	18kDa Antigen gene	ATCGACTGTTGTTTGCGCAAC	[24]
	LP6	18kDa Antigen gene	CCAGCAACCGAAATGTTCGGA	[24]
	LP7	18kDa Antigen gene	TCATAGATGCCTAATCGACTG	[24]
	LP8	18kDa Antigen gene	GGCACATCTGCGGCCAGCA	[24]

There were multiple sets of primers used for the analysis of the multicopy IS*6110* target region: one PCR which used IS*6110* primers [32] for a slightly longer target of 246 bp; another set of primers [33] that amplify a 123 bp product and a third set used in conjunction with the former for a nested PCR [20] which give a 92 bp product. These multiple primer sets were included to address fragmentation. A second genetic region of amplification was used for the detection on *M. tuberculosis*, with primers [34] that target a 203 bp region in the ribosomal protein S12 gene (Table 1). *M. leprae* DNA was amplified using primers for the multicopy RLEP locus [24] as a standard PCR or nested PCR and another set of primers [24] that targets a region of the 18 kDa antigen gene (Table 1).

PCR product (10 µl) was added to 1.0 µl loading dye (MBI Fermentas) and electrophoresed in a 2.0% (w/v) NuSieve (FMC Bioproducts) in TAE (40 mM Tris, 20 mM acetic acid, 1.0 mM EDTA pH 8.0) at 108 volts for 35 minutes. Amplified DNA was visualized by ethidium bromide (0.8 mg/ml) staining exposed to ultraviolet light, recorded with digital imaging equipment (Pharmacia Biotech, ImageMaster).

PCR product was excised from the NuSieve gel and was amplified on a sequencing reaction of 50 cycles of 95°C for 30 seconds, 55°C for 30 seconds and 72°C for 1 minute. The product was directly sequenced on 6% polyacrylamide gel using a radioactive manual sequencing kit (Sequenase Kit, Amersham) and exposed to X-ray film (Kodak) to visualize.

#### Laboratory 2: The Paleo-DNA Laboratory, Lakehead University, Canada

Each sample was mechanically cleaned with a 10% detergent (Tergazyme) solution. They were then rinsed with a 6.0% (w/v) sodium hypochlorite solution followed by 70% ethanol. The teeth were then sprayed with sterilized water and exposed to UV light at 254 nm wavelength and a distance of 30 cm for 20 minutes on all sides. This was done to damage any possible surface contaminants or modern exogenous DNA. To ensure there was no contamination, bone material from inside the bone was removed by a dental pick while the surface of the bone was left intact. The removed bone particles were placed into a mortar and pestle and ground into a fine powder then placed into a sterile tube for extraction. The surface of the tooth samples were removed then pulverized using a steel chamber and Retsch mixer mill and aliquots were placed into sterile 1.5 ml microcentrifuge tubes where they remained dry at room temperature until extraction.

DNA extraction was performed using two methods. The first was a modified Hansen *et al*. [35] extraction method, where approximately 0.3 g of tooth or bone powder was suspended in 400 µl of a proteinase K extraction buffer (0.5 M NaCl, 0.5 M EDTA, 1.0 M tris-HCl pH 8.0, 0.2% SDS, 0.039 M DTT, 0.1 mg/ml proteinase K). Extractions were carried out for 15 hours at 37°C under gentle agitation. Following the initial proteinase K extraction, a phenol-chloroform separation was conducted, followed by purification and concentrating the extracts using ethanol precipitation. The second extraction method used was a modified Boom *et al*. [28] method where approximately 0.3 g of tooth powder was suspended in 500 µl of a guanidinium thiocyanate (GuSCN) extraction buffer (4.0 M GuSCN, 0.1 M tris-HCl pH 6.4, 0.02 M EDTA pH 8.0, 1.3% triton X-100) for 15 hours at 56°C under gentle agitation. Following this extraction, the samples were purified using 20 µl of a silica suspension, which was then washed with 500 µl of working wash buffer (10 mM tris-HCl pH 7.5, 50 mM NaCl, 1.0 mM EDTA, 50% EtOH) and 150 µl of 100% ethanol. The samples were then dried with the DNA bound to the silica until amplification was performed. When required they were resuspended in sterilized water and incubated at 56°C for at least 1 hour. An additional purification was found to remove the inhibition and was conducted on all the samples using Micro Bio-spin P-30 size exclusion chromatography columns (BIO-RAD Laboratories)[36].

The amplification of fragments within HV1 and HV2 of the mitochondrial genome, *M. tuberculosis*, *M. leprae* and the amelogenin and alphoid repeats within the nuclear genome were performed (Table 1). The mitochondrial hypervariable regions were amplified with multiple sets of primers between 100 bp and 350 bp. These combinations of primers were mt15975F and mt16322R (347 bp), mt16190F and mt16420R (230 bp), mt1F or mt15F and mt279R (278 bp or 264 bp respectively) and mt155F and mt439R (284 bp). The *M. tuberculosis* specific primer sets included the INS.1 and the INS.2 and the S12.F and the S12.R primer pairs. The *M. leprae* specific primers were usually performed with the LP1 and LP2 and the LP5 and LP6 primers. Sometimes the nested PCR was performed using the LP3 and LP4 and the LP7 and the LP8 primers. The amelogenin primers were used to identify the sex of the remains along with the alphoid repeats, however the amelogenin is an amplified fragment length product (AFLP) where the alphoid repeats were genetic target of the X or the Y chromosome specifically.

The PCRs were prepared using 1X PCR Buffer, 2.0 mM MgCl_2_, 200 µM dNTPs (Invitrogen), 0.2 µM of each primer, and 1 unit of platinum *Taq* DNA polymerase (Invitrogen). The remaining volume was created using sterilized water and DNA template to total a 25 µl reaction. The amplification cycling parameters were as follows: 94°C for 3 minutes, followed by 45 cycles of 94°C for 30 seconds; 60°C for 1 minute; and 72°C for 2 minutes. PCR products were then analyzed using 6% polyacrylamide gel electrophoresis (PAGE) and stained with ethidium bromide (0.8 mg/ml). Prior to sequencing, the PCR products were purified using the QIAquick PCR purification kits (QIAGEN).

DNA fragments were then sequenced using the ABI Big Dye® Terminator v3.1 cycle sequencing kit (Applied Biosystems) according to the manufacturer's protocol. Forward and reverse sequencing reactions were conducted for all PCR products. Cycling parameters used for sequencing reactions were as follows: 35 cycles of 94°C for 30 seconds; 50°C for 15 seconds; and 72°C for 4 minutes. Sequences were then analyzed using an ABI PRISM 3100 Genetic Analyzer (Applied Biosystems). The sequences were aligned using the BioEdit Sequence Alignment Editor (Tom Hall, Ibis Therapeutics). They were aligned and compared using the revised Cambridge reference sequence and the corresponding *M. tuberculosis* and *M. leprae* targets to determine the presence of polymorphisms [37], [38].

The Paleo-DNA Laboratory is a dedicated ancient DNA facility with stringent precautions and protocols employed for the analysis of degraded DNA. The facility has separated pre-PCR, post-PCR and PCR areas all with separate air handling systems. The pre-PCR area (clean laboratory) is further divided into separate rooms for each analytical task (reagents preparation, samples preparation and PCR preparation) and each work station has dedicated equipment. The researcher wears full body tyvek suits, mask, hoods, booties, eye protection, hair nets underneath and gloves including additional booties, sleeves and gloves over the top of the primary layer. They enter the clean laboratory through an air shower to remove particulates and debris. All reagents, consumables and implements are sterilized in a separate dedicated room (reagent preparation) where detergent soaks, bleach soaks, sonication, baking, autoclaving and cross-linking are used for sterilization. All work surfaces and equipment are cleaned before and after use, daily and the entire laboratory is sterilized weekly. Laboratory controls are taken every month, where all the surfaces are swabbed and analyzed for contamination. The Paleo-DNA Laboratory also carries out forensic case work and follows strict standard operating procedures (SOPs).

#### Laboratory 3: The Centre for Infectious Diseases and International Health, University College London, UK

The amplification of the *M. tuberculosis* and *M. leprae* procedure was replicated in London on samples of the SC1 and SC2 individuals. These samples were prepared by crushing in a sterilized mortar and pestle. Extraction negative control tubes were always processed in parallel with tubes containing samples.

A demineralization solution (100 µl) (0.5 M EDTA pH 8.0, 1.0 mg/ml proteinase K) was added to approximately 25 mg of sample in a sterile 1.5 ml eppendorf tube containing glass beads and mixed, on a mini bead beater, before incubation at 56°C overnight. Lysis buffer L6 [28] was then added (250 µl), the tube vortexed and incubated at 37°C for 2 hours followed by a vortex mix and centrifugation (1 minute at 13 000 g). The supernatant was placed in another sterile tube with 25 µl of silica suspension (12% SiO_2_ w/v, pH 2.0) vortexed and agitated for 2 hours, to allow cell lysis and binding of DNA to the silica.

Samples were resuspended, centrifuged for 1 minute at 13 000 g and the supernatant discarded. The silica was washed with 100 µl washing buffer L2 [28], then washed twice with 70% (v/v) ethanol at −20°C, and once with acetone at −20°C. Tubes were drained on clean, absorbent paper and dried in a 56°C heating block. DNA was eluted with 50 µl of sterile water (Sigma) and incubation at 56°C for 30 minutes. This was repeated once then the eluates were pooled and stored at −20°C.

A nested PCR was used to detect *M. tuberculosis* by targeting the repetitive fragment in the genome of the *M. tuberculosis* complex, the insertion sequence IS*6110*. The primers and method are described by Taylor *et al.* [20], with amplification of a 123 bp region of IS*6110* using primers P1 and P2 (Table 1) [33]. The second set of primers (IS3 and IS4) amplifies an internal 92 bp product (Table 1). Amplification of the RLEP regions in *M. leprae* was performed both as standard PCR and as a nested PCR [24] with amplification of a 129 bp product using the outer primers LP1 and LP2 (Table 1) which can be nested to a 99 bp product in the second round of amplification using LP3 and LP4 (Table 1).

Double strength prealiquoted PCR mix from ABgene® (Epsom, UK) was used and a final volume of 50 µl (75 mM tris-HCl pH 8.8, 20 mM (NH_4_)_2_SO_4_, 1.5 mM MgCl_2_, 0.01% (v/v) tween 20, 200 µM of each nucleotide (dATP, dCTP, dGTP and dTTP), 0.5 µM of each primer; and 1.25 units *Taq* DNA polymerase) The primer pair (2.0 µL), BSA (2 mM), and DNA preparation (5.0 µl) were added to each tube. A control tube with sterile water (Sigma) in place of template was always included.

Amplification consisted of initial denaturation at 94°C for 1 minute and 25 cycles of: 40 seconds at 94°C, annealing at 1 minute at 68°C (IS*6110*) or 58°C (RLEP) and 20 seconds at 72°C, followed by 1 minute at 72°C. For the nested PCR 0.5 µl of product, and 2.0 µl of the IS3/IS4 primer pair were added to each reaction tube and the volume made up to 50 µl. Amplification in both second stage PCRs followed the first protocol, with primer annealing at 58°C.

PCR product (7 µl) was added to 3 µl loading buffer (Sigma) and electrophoresed in a 3.0% (w/v) NuSieve 3∶1 agarose gel (FMC Bioproducts, Flowgen) in TBE buffer (90 mM tris-borate, 2 mM EDTA) or TAE buffer (40 mM Tris-acetate, 1.0 mM EDTA) at 8.8 volts cm^−1^for 80 minutes. Amplified DNA was visualized by ethidium bromide staining exposed under UV light and was recorded with a Polaroid camera.

PCR product (8.15 µl) was mixed with 4 µl loading buffer and separated on a gel as above, by electrophoresis with TAE buffer. The selected band (150–250 mg) was removed from the gel with a sterile scalpel blade into a sterile 0.5 ml tube. DNA was extracted from the gel slices using a MERmaid™ spin kit for samples 10–200 bp (Anachem, UK). The purified product was then sequenced by MWG.BIOTECHAG (Ebersberg, Germany).

Stringent precautions were taken to avoid contamination. Sterile reagents, tubes and plugged tips were used and clean protective clothing worn, with frequent glove changes. A separate room with a different set of pipettes was used for handling PCR product. Pipettes were washed with hydrochloric acid rinsed with ultrapure water and dried with 70% (v/v) ethanol before use. Surfaces were cleaned with neat household liquid detergent, rinsed and dried as above.

## Results

Sequences were generated from the analysis of all the Tomb of the Shroud samples for the HV1 and HV2 of the human mtDNA. The most consistent sequence amplified was 191 bp between mitochondrial nucleotide position (np) 16210 and np16401 (Table 2). The mtDNA profiles from these sequences were analyzed by determining the polymorphisms that occur between these sequences and the revised Cambridge Reference Sequence (Table 3). Polymorphisms in this region will be identical in most maternally related individuals enabling the individuation and maternal relatedness of remains to be interpreted from a familial sample population (Table 4). However the target region is small and thus errors could be introduced in the identification of relationship amongst a random sample population. This mtDNA analysis has identified two mtDNA profiles that are shared between two individuals and one mtDNA profile that is shared between three individuals, suggesting a number of maternal relationships within the individuals from the Tomb of the Shroud (Table 4). The samples were also analyzed to identify their biological sex using the amelogenin gene and the X and Y chromosome specific alphoid repeats (Table 5). All extraction and amplification controls were negative for mtDNA with the primers used in this study. The archaeologist's DNA was characterised for reference to eliminate possible contamination, however neither contamination nor the archaeologist's DNA was found in any of the results.

**Table 2:** Mitochondrial DNA sequence alignment of individuals from the Tomb of the Shroud.

rCRS 16201	CAAGCAAGTA CAGCAATCAA CCCTCAACTA TCACACATCA ACTGCAACTC
Individual SC1	.......... .......... C......... ..........
Individual SC2	.......... .......... .......... ..........
Individual SC2	.......... .......... .......... ..........
Individual SC2	.......... .......... .......... ..........
Individual SC4	.......... .......... .......... ..........
Individual SC7	.......... .......... .......... ..........
Individual SC7	.......... .......... .......... ..........
Individual SC7	.......... ..T....... .......... ..........
Individual SC9	.......... .......... .......... ..........
Individual SC17	.......... ..T....... .......... ..........
Individual SC17	.......... .......... .......... ..........
rCRS 16251	CAAAGCCACC CCTCACCCAC TAGGATACCA ACAAACCTAC CCACCCTTAA
Individual SC1	.......... T......... .......... .......... ..........
Individual SC2	.......... .......... .......... .......... ..........
Individual SC2	.......... .......... .......... .......... ..........
Individual SC2	.......... .......... .......... .......... ..........
Individual SC4	.......... .......... .......... .......... .T........
Individual SC7	.......... .......... .......... .......... .T........
Individual SC7	.......... .......... .......... .......... .T........
Individual SC7	.......... .......... .......... .......... .T........
Individual SC9	.......... .......... .......... .......... ..........
Individual SC17	.......... .......... .......... .......... .T........
Individual SC17	.......... T......... .......... .......... ..........
rCRS 16301	CAGTACATAG TACATAAAGC CATTTACCGT ACATAGCACA TTACAGTCAA
Individual SC1	.......... .......... .......... .......... ..........
Individual SC2	.......... .......... .......... .......... ..........
Individual SC2	.......... .......... .......... .......... ..........
Individual SC2	.......... .......... .......... .......... ..........
Individual SC4	.......... .......... .......... .......... ..........
Individual SC7	.......... .......... .......... .......... ..........
Individual SC7	.......... .......... .......... .......... ..........
Individual SC7	.......... .......... .......... .......... ..........
Individual SC9	.......... .......... .......... .......... .....C....
Individual SC17	.......... .......... .......... .......... ..........
Individual SC17	.......... .......... .......... .......... ..........
rCRS 16351	ATCCCTTCTC GTCCCCATGG ATGACCCCCC TCAGATAGGG GTCCCTTGAC
Individual SC1	.......... .......... .......... .......... ..........
Individual SC2	.......... .......... .......... .......... ..........
Individual SC2	.......... .......... .......... .......... ..........
Individual SC2	...T...... .......... .......... .......... ..........
Individual SC4	.......... .......... .......... .......... ..........
Individual SC7	.......... .......... .......... .......... ..........
Individual SC7	.......... .......... .......... .......... ..........
Individual SC7	.......... .......... .......... .......... ..........
Individual SC9	.......... ..G....... .......... .......... ..........
Individual SC17	.......... .......... .......... .......... ..........
Individual SC17	...T...... .......... .......... .......... ..........
rCRS 16401	CACCATCCTC CGTGAAATCA ATATCCCGCA CAAGAGTGCT ACTCTCCTCG
Individual SC1	.......... ..........
Individual SC2	
Individual SC2	.......... ..........
Individual SC2	.......... ..........
Individual SC4	.......... ..........
Individual SC7	
Individual SC7	...
Individual SC7	.......... ..........
Individual SC9	
Individual SC17	.......... ..........
Individual SC17	.......... ..........

**Table 3:** MtDNA polymorphisms and haplotype of individuals from the Tomb of the Shroud.

Individual	16223	16231	16261	16292	16346	16354	16363	MtDNA Type	Haplotype
**SC1**		C	T					1	
**SC2**								2	H or V
**SC2**								2	H or V
**SC2**						T		3	H
**SC4**				T				4	T3
**SC7**				T				4	T3
**SC7**				T				4	
**SC7**	T			T				5	W
**SC9**					C		G	6	
**SC17**	T			T				5	W
**SC17**			T			T		7	H or J1

**Table 4:** Individual characterisation of skeletal remains and possible relatedness from the Tomb of the Shroud.

Individual	mtDNA Type	Sex	Possible Relationships
**SC1**	1	Adult Male	Unrelated
**SC2**	2	Adult Female	Maternally related to one other individual
**SC2**	2	Infant Indeterminate	Maternally related to one other individual
**SC2**	3	Infant Indeterminate	Unrelated
**SC4**	4	Adult Male	Maternally related to two other individuals
**SC7**	4	Juvenile Female	Maternally related to two other individuals
**SC7**	4	Sex indeterminate	Maternally related to two other individuals
**SC7**	5	Adult Male	Maternally related to one other individual
**SC9**	6	Adult Male	Unrelated
**SC17**	5	Adult Female	Maternally related to one other individual
**SC17**	7	Sex Indeterminate	Unrelated

**Table 5:** The archaeological samples analyzed from the Tomb of the Shroud (SC) and the molecular results for each genetic target.

Sample Tested	Description	Haplo	mtDNA				Sexing			*M. tuberculosis*		*M. leprae*	
			1^st^	2^nd^	3^rd^	4^th^	Amel	ARX	ARY	S12	IS6110	RLEP	18kDa
**SC1 T1^a^**	Tooth		+	+	+	−	+	−	+	+	+	+	+
**SC1 B1^a^**	Bone fragment		−	−	−	−	−	−	−	−	−	−	−
**SC1 B2^a^**	Bone fragment		+	+	+	−	−	−	−	−	−	−	−
**SC1 B3^a^**	Phalange		−	+	−	−	−	−	−	−	−	−	−
**SC1 B4^ab^**	Bone fragment		+	+	+	−	−	+	+	+	+	+	+
**SC1 B5^ab^**	Phalange		−	+	−	−	−	−	−	+	+	+	+
**SC1 F1**	Textile (white fibers)		−	−	−	−	−	−	−	−	−	−	−
**SC1 F2**	Textile (brown fibers)		−	−	−	−	−	−	−	−	−	−	−
**SC1 F3**	Textile (wool fibers)		−	+	−	−	−	−	−	+	+	−	−
**SC1 H1**	Human hair		−	+	+	−	−	−	−	−	−	−	−
**SC2 T1^ab^**	Adult molar tooth	H, V	−	+	+	−	−	+	−	+	+	−	−
**SC2 T2^b^**	Infant tooth	H, V	−	+	−	−	−	−	−	−	+	−	−
**SC2 T3^a^**	Infant tooth	H	+	+	−	−	−	−	−	−	−	−	−
**SC2 B1**	Infant bone fragment	H	−	+	−	−	−	−	−	−	−	−	−
**SC4 T1**	Adult tooth crown	T3	−	+	−	−	−	−	+	−	−	−	−
**SC4 T2^a^**	Adult tooth	T3	+	+	+	+	+	+	+	−	−	−	−
**SC7 T1^a^**	Juvenile incisor piece	T3	−	+	−	−	−	+	+	−	−	−	−
**SC7 T2^a^**	Small molar (broken)	W	+	+	+	−	+	+	−	−	−	−	−
**SC7 B1^a^**	Phalange		−	−	−	−	−	−	−	−	+	−	−
**SC7 B2**	Small phalange	W	−	+	−	−	−	−	−	−	−	−	−
**SC9 T1^a^**	Adult male canine		+	+	+	−	+	+	+	−	−	−	−
**SC17 T1^a^**	Premolar tooth	W	+	+	+	−	+	+	−	−	−	−	−
**SC17 T2^a^**	Adult molar crown	H, J1	−	+	+	−	−	−	−	−	−	−	−
***SC1 R***	*Soil sample*		−	−	−	−	−	−	−	−	−	−	−
***SC1 RW***	*Soil sample*		−	−	−	−	−	−	−	−	−	−	−
***SC1 LW***	*Soil sample*		−	−	−	−	−	−	−	−	−	−	−

The mitochondrial DNA amplification is represented as; 1^st^ mt15975F-mt16322R; 2^nd^ mt16190F-mt16420R; 3^rd^ mt1F or mt15F-mt279R; and 4^th^ mt155F-mt429R. a – indicates samples replicated at the Paleo-DNA Laboratory, b – indicates samples replicated at UCL (only mycobacterial results replicated), + indicates a positive result, - indicates a negative result. SC - corresponds to the Tomb of the Shroud loculus location numbers from the original tomb plan (Figure 1), T - corresponds to Tooth, B - corresponds to Bone and F - corresponds to Textile.

Skeletal and textile remains from numerous loculi and environmental samples were examined and analyzed for the presence of the *M. tuberculosis* DNA. A fragmented bone with what may have been a non-specific pathological lesion was discovered in a collection of archaeological material from the chest region of the shrouded individual's loculus. No other osteological indications of disease were observed from skeletal fragments. Randomly selected bone fragments were genetically analyzed. A phalange, assorted skeletal elements, textile samples and the pulp of a molar tooth from the loculus SC1 (Figure 1) individual were all positive for *M. tuberculosis* (Table 5). The positive amplification of *M. tuberculosis* obtained from two samples of the SC1 individual was independently replicated in the London and Thunder Bay laboratories. Teeth from two infants in the small *ossilegium* niche (SC2 T2 and SC2 T3) in the northern wall of the lower chamber (Figure 1) were also positive for *M. tuberculosis* (Table 5). No osseous bone changes were observed amongst these remains, although infants rarely survive to an age where *M. tuberculosis* lesions are evident in the skeletal material.

The detection method for the *M. tuberculosis* complex DNA was based on the extraction and subsequent amplification of the IS*6110* repeat element and the S12 ribosomal protein gene. All amplifications obtained from archaeological samples were identical to the NCBI Genbank sequence for IS*6110* (sequence X17348, bases 788–863) and the S12 protein (sequence X70995 in the NCBI database) (Table 6 and 7). The *M. leprae* DNA was detected by amplification of the 18 kDa antigen gene (sequence MSGANT18K in the NCBI database) and the RLEP repetitive elements (sequence X17153 in the NCBI database) (Table 8 and 9). Amplification of the mitochondrial DNA from these same samples and both positive and negative results for pathogenic DNA from these tombs in addition to logical and expected results for the haplogroups identified supports authentic results. All extraction and amplification controls were negative for the pathogenic DNA. No positive PCR results were obtained from any of the environmental archaeological site control samples for the presence of *M. tuberculosis* DNA.

**Table 6:** Alignment of *M. tuberculosis* IS*6110* repeat element and sequence data from SC1 exhibiting complete sequence homology.

*M. tuberculosis*	CGTGAGGGCATCGAGGTGGCCAGATGCACCGTCGAACGGCTGATGACCAA
SC1 concensus	..................................................
SC2 concensus	..................................................
SC2 concensus	..................................................
SC7 concensus	..................................................
*M.tuberculosis*	ACTCGGCCTGTCCGGGACCACCCGCGGCAAAGCCCGCAGGACCACGATCG
SC1 concensus	..................................................
SC2 concensus	..................................................
SC2 concensus	..................................................
SC7 concensus	..................................................
*M.tuberculosis*	CTGATCCGGCCACAGCCCGTCCCGCCGATCTCGTCCAGCGCCGCTTCGGA
SC1 concensus	..................................................
SC2 concensus	..................................................
SC2 concensus	..................................................
SC7 concensus	..................................................
*M.tuberculosis*	CCACCAGCACCTAACCGGCTGTGGGTAGCAGACCTCACCTATGTGTCGAC
SC1 concensus	..................................................
SC2 concensus	..................................................
SC2 concensus	..................................................
SC7 concensus	..................................................
*M.tuberculosis*	CTGGGCAGGGTTCGCCTACGTGGCCTTTGTCACCGACGCCTACGTCGC
SC1 concensus	................................................
SC2 concensus	................................................
SC2 concensus	................................................
SC7 concensus	................................................

* Underlined sequence denotes the primer binding region.

Aligned to the IS6110 sequence from Genbank, accession number X17348.

**Table 7:** Alignment of *M. tuberculosis* gene for ribosomal protein S12 with sequence data from SC1.

*M.tuberculosis*	TCGTCGGGACAAGATCAGTAAGGTCAAGACCGCGGCTCTGAAGGGCAGCC
SC1	..................................................
*M. tuberculosis*	CGCAGCGTCGTGGTGTATGCACCCGCGTGTACACCACCACTCCGAAGAAG
SC1	..................................................
*M. tuberculosis*	CCGAACTCGGCGCTTCGGAAGGTTGCCCGCGTGAAGTTGACGAGTCAGGT
SC1	..................................................
*M. tuberculosis*	CGAGGTCACGGCGTACATTCCCGGCGAGGGCCACAACCTGCAGGAGCACT
SC1	..................................................
*M. tuberculosis*	CGAT
SC1	....

* Underlined sequence denotes the primer binding region.

Aligned to the S12 sequence from Genbank, accession number X70995.

**Table 8:** Alignment of *M. leprae* 18 kDa gene region with sequence data from SC1.

*M. leprae*	ATCGACTGTTGTTTGCGCAACAAGCTAAGCACGAAGCTAAGCACGCCGCT
*SC1*	..................................................
*M. leprae*	GCGGTCAAAAGCCCGTCTTAGCCATGGATCTTTAAAGTATCCGAACATTT
*SC1*	..................................................
*M. leprae*	CGGTTGCTGG
*SC1*	..........

* Underlined sequence denotes the primer binding region.

Aligned to the 18 kDa gene sequence from Genbank, accession number MSGANT18K.

**Table 9:** Alignment of *M. leprae* RLEP gene region with sequence from SC1.

*M. leprae*	TGCATGTCATGGCCTTGAGGTGTCGGCGTGGTCAATGTGGCCGCACCTGA
*SC1*	..................................................
*M. leprae*	ACAGGCACGTAAAAGTGCACGGTATAACTATTCGCACCTGATGTTATCCC
*SC1*	..................................................
*M. leprae*	TTGCACCATTTCTGCCGCTGGTATCGGTG
*SC1*	.............................

* Underlined sequence denotes the primer binding region.

Aligned to the RLEP gene sequence from Genbank, accession number X17153.

Genetic detection of *M. leprae* exclusively from the SC1 shroud loculus individual was also successful. Results have been successfully replicated in both London and Thunder Bay laboratories using multiple controls, including environmental soil samples and tomb wall surface scrapings. All extraction and amplification controls were negative for pathogenic DNA. No positive PCR results were obtained from any of the environmental archaeological site control samples for the presence of *M. leprae* DNA. The use of stringent ancient DNA protocol, multiple genetic targets with 100% sequence homology to pathogenic targets and a cognitive approach to authentication of mitochondrial DNA sequence analysis, sex and familial relatedness results was used to ensure authenticity of our ancient DNA results [39].

## Discussion

MtDNA analyses have been performed on the primary loculi (kokhim) burials and secondary skeletal (ossilegium) deposits from the first-century C.E. Tomb of the Shroud (SC) found in Jerusalem to clarify the familial relationship of the tomb occupants. There were ten samples analyzed from the Tomb of the Shroud loculus, SC1 (Figure 1). These included one tooth sample, five bone samples, three textile sample (some with hair associated) and one hair sample. The analysis included hair samples from the loculus and hair that was found attached to some of the textile samples. All of these analyzed samples produced the same human mtDNA profile (191 bp) and the individual was identified to be male. It was deemed important to test numerous samples of bone and the tooth, due to the disturbed nature of the biological remains in the shroud loculus, and a prior expectation that these loculi were reused. The mtDNA results indicate that there was only one individual within the loculus (SC1) that also contained the shroud. The analysis of the hair attached to the textile and the hair itself, confirmed the context of the skeletal material associated with the textile, referred to as a burial shroud that was found in this loculus.

There were four samples tested from the Tomb of the Shroud SC2 location (i.e. the ossilegium niche in the north wall of the lower chamber) (Figure 1). These were identified as a female and two infants for which their sex was not identified. The female and one of the infant samples had mtDNA type 2, and the second infant had mtDNA type 3. The fourth sample was found to have the same mtDNA type and morphological size as one of the three individuals concluded to be a replicate sample.

There were two samples analyzed from the Tomb of the Shroud SC4 location (Figure 1). Both these samples produced the same mtDNA type 4 and were both found to be male. These two samples are thus more likely to be from the same individual.

There were four samples tested from the SC7 location (Figure 1). These were identified as one male and one female with different mtDNA types (mtDNA type 5 and type 4 respectively). However, the third sample contained a mtDNA sequence that matched one of the previous samples (mtDNA type 4). These two samples are more than likely to be from the same individual rather than that of two related persons. However, the genetic sex analysis of this sample was indeterminate and unable to clarify this issue. The fourth sample tested from SC7 did not produce a reliable mtDNA sequence but produced an IS6110 sequence and was not included in the study thereafter.

There was only one sample tested from the SC9 location (Figure 1). This sample was identified as a male with mtDNA type 6, which did not match any other member of the tomb that was tested. From SC17 there were two samples tested (Figure 1). These produced one female sample and one sex indeterminate sample. These samples both had different mtDNA types, the female mtDNA type 5 and the other mtDNA type 7. The type 7 sample did not match any other sample in the tomb.

### Relatedness Analysis

From this analysis there have been identified two maternally unrelated male individuals (both adults from SC1 and SC9), two maternally unrelated sex indeterminate individuals (one adult SC17 and one infant SC2) and two maternally related pairs of individuals (adult female SC2 and infant SC2 and adult female SC17 and adult male SC7). If the third sample from SC7 has the correct provenance, then this is potentially a group of three maternally related individuals (adult male SC4, juvenile female SC7 and sex indeterminate SC7).

### Textile

Mitochondrial DNA analysis confirmed the locks of hair were contextually associated with the skeletal remains found in loculus SC1. The mtDNA analysis also confirmed the textile was associated with the hair found in loculus SC1 and that the textile was draped or shrouded over the hair and head region. It was also identified that the textile was present adhering to biological breakdown products on the floor of the loculus, the entire length of the body of the individual in loculus SC1.

The haplotype designations for mtDNA analyses are population categorizations that have been used extensively in the past to infer population movements [10]. The haplotypes for the Tomb of the Shroud individuals are commonly distributed throughout the North of Africa and the Middle East through to Eastern Europe. The tomb occupants exhibit a number of different maternal influences. The maternal relatedness of these individuals supports inferences that this Akeldama site was a family tomb which was in use in the first-century C.E.

A selection of osteological elements from the Tomb of the Shroud in the Hinnom valley has been genetically analyzed to investigate the presence of disease in this unusual occurrence of a primary burial of a shrouded individual walled off, using rock and plaster, within the tomb. This is a unique archaeological discovery, suggesting it might have been intentionally kept sealed by family members who feared the visual aspects of disease.

Pathology was suspected in the anthropological examination of human remains from SC1 (made by D. Sklar). A bone with an advanced lesion was later identified in a collection of bones from the chest region of the SC1 individual. The presence of this lesion supports the genetic identification of *M. tuberculosis* bacilli in all the skeletal elements analyzed and the severe nature of the infection. The positive results from the textile sample could be produced from the infected blood and biological material that was found to be adhered to the textile. There were no bone lesions identified in remains from SC2, although infants rarely survive long enough for tuberculosis bone lesions to form.

The mitochondrial DNA profile was not obtained for one of the SC7 samples for which we are unable to resolve the provenance. The differential preservation of mitochondrial and pathogenic DNA has been noted in many cases as the amplification intensity of pathogenic DNA, is far greater than the amplification intensity of mitochondrial DNA suggesting a higher concentration of pathogenic DNA template.

The identification of *M. tuberculosis* from an individual approximately 2,000 years ago is consistent with our current understanding of *Mycobacterium* infection and transmission between humans and in humans and domesticated animals [40]. *Mycobacterium bovis* is a distinct pathogenic species in bovids, appearing with domestication in the Middle East, is not common in human populations, and has only once been confirmed in the past by biomolecular analysis of archaeological material [41]. The first pathogenic *Mycobacterium* to arise and its host population is a contentious issue and may be resolved with future molecular research on archaeological samples and the hunt for a progenitor or an intermediate *M. tuberculosis/M. bovis* species [41], [42], [43].

Tuberculosis is an opportunistic pathogenic disease common and generally fatal in antiquity throughout Europe and the Middle East. The shrouded individual (SC1) and two infants (SC2) were genetically confirmed to contain the DNA from *M. tuberculosis*. Identification of *M. tuberculosis* from the tooth pulp of these individuals suggests that this pathogen was in the bloodstream and therefore throughout the body as miliary tuberculosis and all three individuals would have almost certainly have died from a disseminated bacterial infection.

The three *M. tuberculosis* positive individuals do not appear to be maternally related, they may however be paternally related (this will be investigated in future Y chromosome analyses). The skeletal remains in the wall niche were those of the two infants with tuberculosis and a maternally related adult female, possibly the mother. Future genetic work using SNP typing of the pathogen infecting each individual may identify the epidemiological transmission pattern of this pathogen amongst individuals within this tomb.

There have been few genetically confirmed cases of pathological *Mycobacterium spp.* infections in Israel. Rafi *et al.* [18] published the first molecular identification of leprosy, *M. leprae,* in archaeological samples from Israel, followed by the amplification of *M. leprae* from a Madura foot dating from the Byzantine period [44]. The molecular identification of *M. leprae* from SC1 in the first-century C.E. Tomb of the Shroud is the earliest molecularly confirmed case of leprosy (Hansen's Disease) from Israel. In the Near East leprosy is not generally thought to predate the Hellenistic period and the troops of Alexander the Great (327–326 B.C.E) were thought to be the ones responsible for bringing this deadly disease back from the Far East (specifically India). A recent hypothesis [45] suggests that leprosy may have first appeared in the region not with Alexander but sometime earlier (*circa* 400 B.C.E or later), with diseased young slaves conveyed from India to Egypt on cargo ships. More recently a review of the origins and spread of leprosy has identified an East African origin with various routes of migration out of Africa into the Middle East and beyond [46]. Leprosy was, and still is associated with enormous social stigma, and individuals could have been ostracized from their communities. The health of expelled lepromatous individuals is probably compromised, without assistance for food procurement or an income. On infection the individual becomes immuno-compromised and the chance of co-infection by *M. tuberculosis* and other pathogens occurs. However, a number of indications – the location and size of the tomb, the type of textiles used as shroud wrappings, the clean state of the hair – suggest that the shrouded individual was a fairly affluent member of society in Jerusalem and that tuberculosis crossed social boundaries in the first century C.E.

It is interesting to note that the skeletal remains from within the plaster-sealed parts of the tomb (SC1 and SC2) are positive for *M. tuberculosis* and one of them for both *M. tuberculosis* and *M. leprae*. There appears to be a correlation therefore between the presence of disease in the human remains and the isolation of those remains even when these remains had undergone secondary burial (as with the wall niche remains - SC2). Sealing parts of the tomb with white plaster is generally rare in first-century C.E. tombs in Jerusalem. Thus it would suggest that these remains were intentionally sealed. However, as previously mentioned, the individuals from the wall niche undoubtedly underwent secondary burial, meaning that their bones were extracted from a loculus and placed into the niche.

The presence of both *M. tuberculosis* and *M. leprae* DNA is associated with immuno-suppressed individuals, which is a feature of the multi-bacillary or lepromatous form of leprosy. Indeed, in historical times leprosy patients were often reported to have died due to the more aggressive *M. tuberculosis* infection [47], [48], [49]. A secondary co-infection of tuberculosis, in Hansen's patients is found in 25% of modern cases [50], and up to one-third of the world's population is currently infected with tuberculosis (WHO 2007: World Health Organisation: http://www.who.int/tb). The co-infection of leprosy with tuberculosis is often the cause of death as *M. leprae* infection generates a compromised immune system, vulnerable to a subsequent *M. tuberculosis* infection that is still fatal in some parts of the world today. The finding of co-infection in the shrouded individual stimulated us to review all our positive *M. leprae* cases and resulted in a hypothesis being proposed as to why leprosy disappeared from Europe in Medieval times [27].

Due to the highly degraded nature of the skeletal remains from the Hinnom Valley tomb and the disturbed archaeological context of the individuals, molecular sex determination, mtDNA for maternal familial relationships and molecular paleopathology dramatically enhanced the significance of this archaeological site. Three individuals were detected with tuberculosis, all of which were recovered from plaster sealed locations. A co-infection of tuberculosis and leprosy was an unexpected discovery in the remains of the shrouded individual and present a significant and unique anthropological example of contagious disease in the past.

### Conclusion

The Tomb of the Shroud suffered from illegal and clandestine digging operations before archaeologists reached the site, and this resulted in the disturbed nature of the degraded human remains and limited the physical anthropological examination. Genetic analysis confirmed the archaeologist's interpretation of this tomb as a family tomb containing a number of generations and provided important information on the bioarchaeology not available through skeletal analysis.

The presence of *M. tuberculosis* and *M. leprae* in these individuals within the Tomb of the Shroud is significant for the geographical and temporal distribution of tuberculosis and leprosy in the past. It confirms the presence of tuberculosis using molecular methods and is the oldest molecular confirmation of both *M. tuberculosis* and *M. leprae* in Jerusalem. Co-infection present a plausible explanation for the unusual and extreme lengths people went to in order to contain what was recognized as a seriously debilitating affliction. This analysis found that the shroud loculus individual and two infants from the same tomb suffered from *M. tuberculosis* infection. The prevalence of such a highly contagious disease, particularly for immuno-compromised individuals with leprosy is not unexpected with inadequate sanitation and demonstrates the significant impact social diseases such as tuberculosis had on society from the low socioeconomic groups up to the more affluent families, such as Tomb the Shroud in first-century Jerusalem.

Molecular archaeology and the identification of pathogenic DNA contribute greatly to the confirmation of pathologies and direct identification of disease in fragmented and degraded material such as this tomb. The molecular identification of tuberculosis in the Jerusalem tomb is a significant contribution to the archaeological interpretation of this site and suggests a reason for the plaster sealing of the loculus containing the shrouded man and for the additional individuals sealed in the wall niche. Molecular pathology adds a new dimension to the archaeological exploration of disease in ancient times, especially in regard to the remains of infants where there is an absence of bone pathologies. The discovery and successful genetic analyses of unique archaeological sites such as Tomb of the Shroud poses great promise for future investigations into the host-pathogen relationships and evolution, geographic distribution and epidemiology of disease and social health in the past.
