# Correction: Molecular Exploration of the First-Century *Tomb of the Shroud* in Akeldama, Jerusalem

**DOI:** 10.1371/annotation/32ada7b9-3772-4c08-9135-b5c0933f0b5e

**Published:** 2010-04-29

**Authors:** Carney D. Matheson, Kim K. Vernon, Arlene Lahti, Renee Fratpietro, Mark Spigelman, Shimon Gibson, Charles L. Greenblatt, Helen D. Donoghue, Boaz Zissu

Dr. Boaz Zissu was mistakenly omitted from the author byline. He should be listed as the ninth author and is affiliated with Martin (Szusz) Department of Land of Israel Studies and Archaeology, Bar Ilan University, Israel. His contribution included excavating materials for analysis.

